# ABO-identical matching has no superiority in long-term survival in comparison to ABO-compatible matching in lung transplantation

**DOI:** 10.1186/s13019-019-0846-6

**Published:** 2019-01-28

**Authors:** Mohammed Fakhro, Hillevi Larsson, Malin Malmsjö, Lars Algotsson, Sandra Lindstedt

**Affiliations:** 1 Dept. of Cardiothoracic Surgery, Lund University Hospital, Lund University, 221 85 Lund, Sweden; 2 Pulmonary Medicine, University Hospital, Lund University, Lund, Sweden; 3 Ophthalmology, University Hospital, Lund University, Lund, Sweden; 4 Thoracic Intensive Care and Anesthesia, Skåne University Hospital, Lund University, Lund, Sweden

**Keywords:** Lung transplantation, Survival, Patient outcome, ABO-blood group

## Abstract

**Background:**

Even though identical blood group matching between recipient and donor is preferred, it is still not clear by how much this improves the outcome for patients who received a lung transplant (LTx), or whether there is any survival benefit. Earlier studies have yielded ambiguous results and few have investigated long-term survival. The aim of this study is, therefore, to explore the different outcomes of identical and compatible recipient and donor blood group matching to determine whether identical matching is superior (LTx).

**Method:**

Between January 1990 to June 2016, 297 patients underwent primary LTx, 10 patients underwent heart and lung transplantation (HLTx), and 18 patients required re-transplantation (Re-LTx) at Skåne University Hospital in Lund. With a total of 325 transplantations at our center, 262 were ABO-identically matched while 53 were ABO-compatible. For survival analyses, the end-point used was retransplantation-free survival in addition to excluding HLTx (*n* = 10), assessed by Cox regression and Kaplan-Meier.

**Results:**

ABO-compatible patients had a median of 49 days (2–641), and ABO-identical patients had a median of 89 days (1–1717) (*p* = 0.048) on the transplant waiting list. Patients with a limited survival up to 1-year showed significant difference in survival rate for ABO-compatible recipients compared to ABO-identical recipients (*p* < 0.05), however no significant difference was shown in overall survival between the two groups (*p* > 0.05), with the same pattern shown in patients with a limited survival rate up to ten years, emphysema-patients, when excluding single-LTx and patients transplanted before 2005 and after 2005, respectively (*p* > 0.05).

**Conclusion:**

Recipients who received ABO-compatible matched grafts showed a similar survival rate to recipients who received ABO-identical matched grafts in the present study. Cytolomegalovirus and Ebstein Barr Virus mismatch were also identified as risk factors particular among emphysema patients. Since ABO-identical transplantations and ABO-compatible transplantations showed similar results, the present selection-bias of preferring ABO-identical lungs could be adjusted to increase organ allocation. It might also be possible to shorten recipient waiting list time, as an identical match showed over 80% higher time on the waiting list than a compatible, non-identical match.

## Introduction

ABO-identical matching has long been preferred over minor ABO-mismatching (i.e. compatible but non-identical) in transplantation [1, 2] because it was believed to decrease the risk of organ rejection [3–5]. ABO-identical matching has been shown to prevent recipient erythrocyte destruction by the lymphocytes found in the donor organ, ultimately leading to hemolysis and organ failure such as in the kidneys [6]. Matching age, height, and size are established requirements in an organ allocation program to ensure a successful transplantation and optimal post-operative survival [7–10]. Other donor and recipient factors include gender, Cytologmegalovirus (CMV), Ebstein Barr Virus (EBV) and toxoplasma-serology, smoking status, HLA-status, and screening through bronchoscopy and chest X-ray. Studies investigating recipient-donor mismatching of these factors have yielded conflicting results regarding their impact on patients’ long-term survival. Matching these parameters might, therefore, unnecessarily restrict the number of lung transplantations (LTx) that can be performed from an already scarce donor pool [11]. Earlier studies have recommended ABO-matching, but the long-term benefits are unclear, including whether it improves survival.

The relationship between age and identical ABO-matching might also be important. It has been shown in heart transplantation (HTx), for instance, that younger patients have a reduced risk for postoperative infections and a higher risk for organ rejection compared to elderly patients [12]. It has been suggested that this relationship is explained by immunological factors that change with age. There is, however, still no consensus whether recipient age and ABO-matching interact in LTx. This retrospective cohort study aims to better understand the effect of ABO-identical vs. ABO-compatible matching on survival in LTx and to explore additional risk factors.

## Patients and methods

### Data source

Between January 1990 and June 2016, 307 patients underwent LTx at Skåne University Hospital in Lund. Out of these, 197 were double-lung transplanted (DLTx), 100 were single-lung transplanted (SLTx), and 10 patients underwent heart- and lung transplantation (HLTx). The median age for these patients was 52 years with a range of 12–72 years. In terms of gender, 145 were males and 162 females. The major indications for a LTx or HLTx were chronic obstructive pulmonary disease (COPD) (*n* = 74), cystic fibrosis (CF) (*n* = 59), α1-antitrypsin deficiency (AAT1) (n = 59), pulmonary fibrosis (PF) (*n* = 43), pulmonary hypertension (PH) (*n* = 39), and a group deemed as “Others” (*n* = 33), which included bronchiectasis, sarcoidosis, bronchioalveolary cancer, silicosis, and graft-vs-host disease (GVHD).

In addition, 18 patients required re-lung transplantation (Re-LTx). Out of these, 8 were DLTx and 10 SLTx. In terms of gender, 12 were male and 6 female. The major indications for Re-LTx were defined as chronic lung allograft dysfunction (CLAD) (*n* = 15), primary graft dysfunction (PGD) (n = 1), malignancy (n = 1), and mechanical complication (n = 1).

With a total of 325 transplantations (LTx, HLTx, Re-LTx), 262 were ABO-identical while 53 were ABO-compatible. In total, 10 patients had missing data regarding ABO-blood groups.

ABO-compatibly matched LTx groups consisted both of group O donors matching for A, B, AB recipients as well as group A, B donors matching for AB recipients. Examples of situations where an ABO compatible donor was used instead of an ABO identical matching were often due to the scarcity in donors and the individual assessment of each recipient that the risk of waiting additionally for a LTx on the waiting-list would be to great.

### Statistical methods

Data are presented as mean with standard deviation (SD), median with range, or frequency with percentage. Shapiro-Wilks test was used to determine which variables were normally distributed/parametric (mean, SD) vs. non-normally distributed/non-parametric (median, range). Independent (unpaired) student’s t-test was conducted for normally distributed continuous variables while Mann-Whitney U (Wilcoxon rank sum) test was used for non-normally distributed continuous data. Chi-square test or Fisher’s exact test were chosen for analysis of categorical variables. For survival analysis, the end-point used was retransplantation-free survival in addition to excluding HLTx. Cox regression estimates in accordance with Cox proportional hazards model was performed for univariable survival analysis. Survival estimates were displayed in accordance with Kaplan-Meier with Log-rank test to detect significance between survival curves. A *p*-value < 0.05 was considered statistically significant. Statistical analyses were performed using SPSS Version 24.0 (IBM Corp., Armonk, NY, USA).

## Results

### Recipient and donor characteristics

**Fig. 1:**
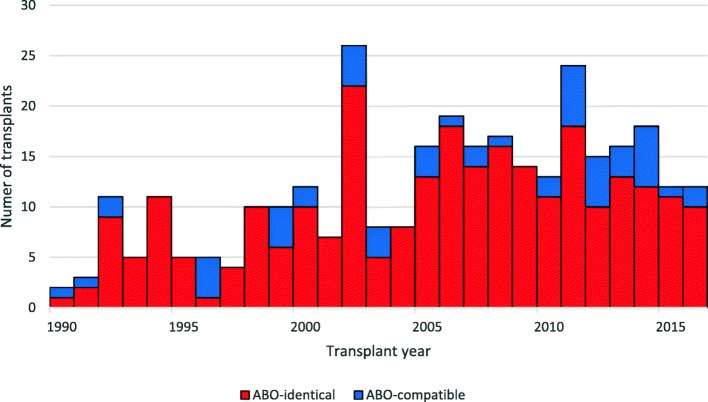
Temporal distribution of all LTx at our single-center stratified into ABO-identical (*N* = 262) and ABO-compatible (*N* = 53) transplants between January 1990 to June 2016. Absolute numbers illustrated (bars)

Distribution in time for ABO-identical and ABO-compatible transplants between January 1990 and June 2016 are illustrated in Fig. 1.

Baseline and clinical characteristics of both recipients and donors are shown in Table 1. A statistically significant difference was found in waiting-list time between ABO-compatible and ABO-identical LTx with 49.0 (2.0–641.0) days and 89.0 (1.0–1717.0) days, respectively (*p* = 0.048).

**Table 1:** Recipient/donor baseline and clinical characteristics of ABO-compatible and ABO-identical LTx

Variables	ABO-compatible (*n* = 53)	ABO-identical (*n* = 262)	*p*-value
Recipient data			
*Recipients major indication*			0.240
COPD	10 (18.9%)	64 (24.4%)	
AAT1	9 (17.0%)	49 (18.7%)	
PH	7 (13.2%)	29 (11.1%)	
CF	15 (28.3%)	43 (16.4%)	
PF	9 (17.0%)	35 (13.4%)	
Others	2 (3.8%)	25 (9.5%)	
Graft failure (Re-LTx)	1 (1.8%)	17 (6.5%)	
CMV serology (pos)	39 (73.6%)	205 (78.2%)	0. 573
EBV serology (pos)	37 (69.8%)	184 (70.2%)	0. 920
Toxoplasma serology (pos)	14 (26.4%)	62 (23.7%)	0. 680
CMV-mismatch	8 (15.1%)	41 (15.6%)	0. 911
EBV-mismatch	5 (9.4%)	17 (6.5%)	0. 448
Toxoplasma mismatch	6 (11.3%)	31 (11.8%)	0. 916
Weight (kg)	64. 3 ± 19. 9	59. 8 ± 12. 5	**0. 032**
Recipient/Donor weight ratio	0. 9 (0. 4–3. 1)	0. 8 (0. 4–1. 6)	**0. 034**
Height (cm)	169. 1 ± 9. 1	168. 6 ± 10. 4	0. 703
Recipient/Donor height ratio	1. 0 (0. 9–2. 4)	0. 9 (0. 8–1. 1)	0. 692
BMI	22. 2 ± 4. 2	20. 9 ± 3. 7	**0. 045**
Male	28 (52.8%)	124 (47.3%)	0. 465
Gender mismatch	16 (30.2%)	90 (34.4%)	0. 523
Age (years)	45. 5 (12. 2–70. 6)	52. 9 (12. 4–72. 0)	0. 159
Recipient/Donor age ratio	0. 97 (0. 30–3. 92)	1. 03 (0. 27–3. 99)	0. 102
Waiting list (days)	49.0 (2. 0–641. 0)	89. 0 (1. 0–1717. 0)	**0. 048**
*Lab values*			
FVC (liters)	2. 0 (0. 7–5. 2)	2. 1 (0. 3–5. 3)	0. 233
FEV1 (liters)	0. 9 (0. 2–2. 6)	0. 8 (0. 2–3. 4)	0. 735
6MWT (%)	39. 4 ± 20. 3	38. 6 ± 19. 4	0. 813
P-ALT (μkat/L)	0. 4 (0. 1–9. 7)	0. 4 (0. 1–1. 6)	0. 128
P-AST (μkat/L)	0.5 (0. 2–10. 0)	0. 4 (0. 2–2. 3)	0. 340
P-creatinine (μmol/L)	66 (22–234)	62 (26–217)	0. 255
Pulm. pressure > 25mmhg	20 (37.8%)	70 (26.7%)	0. 110
*Tx-type*			0. 121
SLTx	14 (26.5%)	86 (32.8%)	
DLTx	36 (67.9%)	157 (59.9%)	
HLTx	2 (3.8%)	2 (0.8%)	
Re-LTx	1 (1.8%)	17 (6.5%)	
SLTx	1 (100%)	9 (52.9%)	
DLTx	0 (0%)	8 (47.1%)	
ATG	35 (66.0%)	172 (65.6%)	0. 908
*Pre-op Life support*			
Mechanical ventilation	2 (3.8%)	12 (4.6%)	0. 795
ECMO	3 (5.7%)	9 (3.4%)	0. 673
Donor data			
CMV serology (pos)	32 (60.4%)	172 (65.6%)	0. 547
EBV serology (pos)	22 (41.5%)	139 (53.1%)	0. 119
Toxoplasma serology (pos)	8 (15.1%)	48 (18.3%)	0. 568
Weight (kg)	70 (19–180)	71 (40–105)	0. 901
Height (cm)	170 (70–198)	170 (150–190)	0. 886
BMI	23. 9 (6. 2–34. 6)	23. 9 (14. 7–35. 9)	0. 999
Male	27 (50.9%)	119 (45.4%)	0. 506
Age (years)	50. 5 (12. 2–71. 4)	47. 0 (7. 8–75. 4)	0. 463

Data are mean (SD), number (%), or median (range). The numbers are based on patients with data available

Entries are bold due to being under 0.05 which are significant results

*COPD* chronic obstructive pulmonary disease, *AAT1* Alpha 1-antitrypsin deficiency, *PH* pulmonary hypertension, *CF* cystic fibrosis; PF, pulmonary fibrosis, *CMV* cytomegalovirus; *EBV* Epstein-barr virus; BMI, body-mass index, *FVC* forced volume vital capacity, *FEV1* forced volume expiratory capacity 1 s, *6MWT* 6-min walking test, *AST* aspartate transaminase; *ALT* alanine transaminase, *SLTx*, single-lung transplantation, *DLTx* double-lung transplantation, *HLTx* heart-lung transplantation, *Re-LTx* re-lungtransplantation, *ATG* anti-thymocyte globulin, *ECMO* extracorporeal membrane oxygenation

No differences between recipient characteristics such as pulmonary function (FVC, FEV1, 6MWT), liver/kidney-status (AST, ALT, creatinine), and pre-operative life support (ECMO or mechanical ventilation) were shown (*p* > 0.05).

Among donor characteristics, no significant differences was found between ABO-identical and ABO-compatible LTx (p > 0.05).

### Mortality

Cause of death during follow-up stratified between ABO-compatible and ABO-identical LTx is illustrated in Table 2. No difference was found in cause of death (rejection, infection, malignancy, or “miscellaneous”) between ABO-compatible and ABO-identical LTx (*p* = 0.795). No statistical significant difference found in cause of death grouped by donor blood group (*p* = 0.902).

**Table 2:** Cause of death after ABO-compatible and ABO-identical transplants in addition to donor blood group

				Donor blood group				
	ABO-compatible *n* = 28 (%)	ABO- identical *n* = 123 (%)	p-value	A *n* = 51 (%)	B *n* = 17 (%)	AB n = 1 (%)	O *n* = 82 (%)	*p*-value
Cause of death								
			0. 795					0. 902
Rejection	6 (21)	34 (27)		12 (24)	4 (24)	1 (100)	23 (28)	
Infection	9 (32)	29 (24)		13 (25)	5 (29)	0 (0)	20 (24)	
Malignancy	4 (15)	18 (15)		9 (18)	3 (18)	0 (0)	10 (13)	
Miscellaneous	9 (32)	42 (34)		17 (33)	5 (29)	0 (0)	29 (35)	

The group called ‘miscellaneous’ is defined as patients with mortality caused by myocardial and cerebral ischaemia, and multiple organ failure such as renal and liver in addition to other causes related to the patient’s old age and individual health status

### Survival estimates

**Fig. 2:**
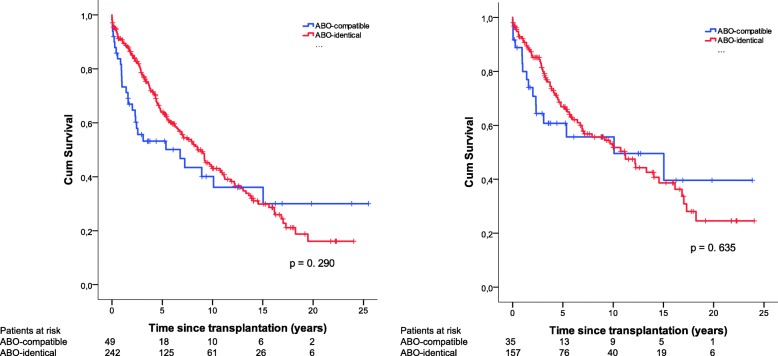
Cumulative retransplantation-free survival for ABO-compatible (*N* = 49) and ABO-identical (*N* = 242) transplants between 1990 and 2016 for the entire cohort excluding heart-lung transplantations (left figure) in addition to ABO-compatible (*N* = 35) and ABO-identical (*N* = 157) LTx when excluding patients that underwent single-lung transplantation (right figure)

Between ABO-identical vs. ABO-compatible LTx for the entire cohort (excluding HLTx) in addition to excluding SLTx, cumulative survival rate estimates at 1-, 5, 10, 15-, and 20-years are illustrated in terms of percentage with an upper/lower 95% confidence interval (CI) (Fig. 2). For the entire cohort between 1990 and 2016 excluding HLTx, ABO-identical matches showed 1-, 5-, 10-, 15-, and 20-year survival rates of 91% (CI 87–95), 64% (CI 58–70), 44% (CI 36–51), 30% (CI 23–38), and 16% (CI 7–25), respectively, compared to patients with ABO-compatible matching at 1-, 5-, 10-, 15-, and 20-year survival rates of 73% (CI 60–86), 53% (CI 39–68), 40% (CI 24–56), 36% (CI 20–51), and 30% (CI 13–47), respectively (*p* = 0.290). In addition to excluding patients that underwent SLTx, no significant difference was found (*p* = 0.635).

**Fig. 3:**
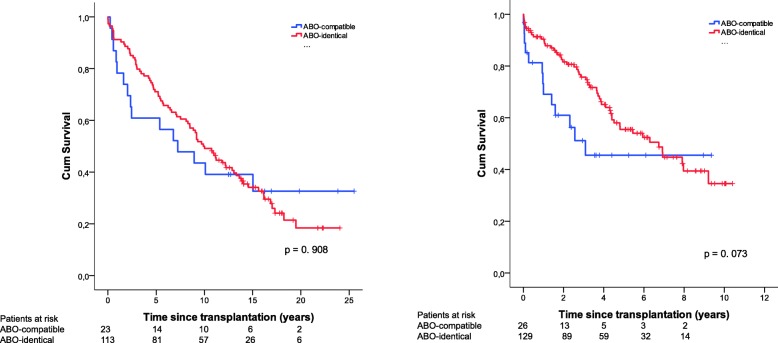
Cumulative retransplantation-free survival for ABO-compatible (*N* = 23) and ABO-identical (*N* = 113) transplants for the period 1990-2005 (left figure) and ABO-compatible (N = 26) and ABO-identical (*N* = 129) transplants for the period 2006-2016 (right figure). No significant difference in survival was observed between the two groups in either of the time periods

Patients that underwent LTx between the periods 1990–2005 and 2006–2016 are shown in Fig. 3. No significant difference in survival was found between ABO-identical and compatible LTx in either time-period (*p* > 0.05).

**Fig. 4:**
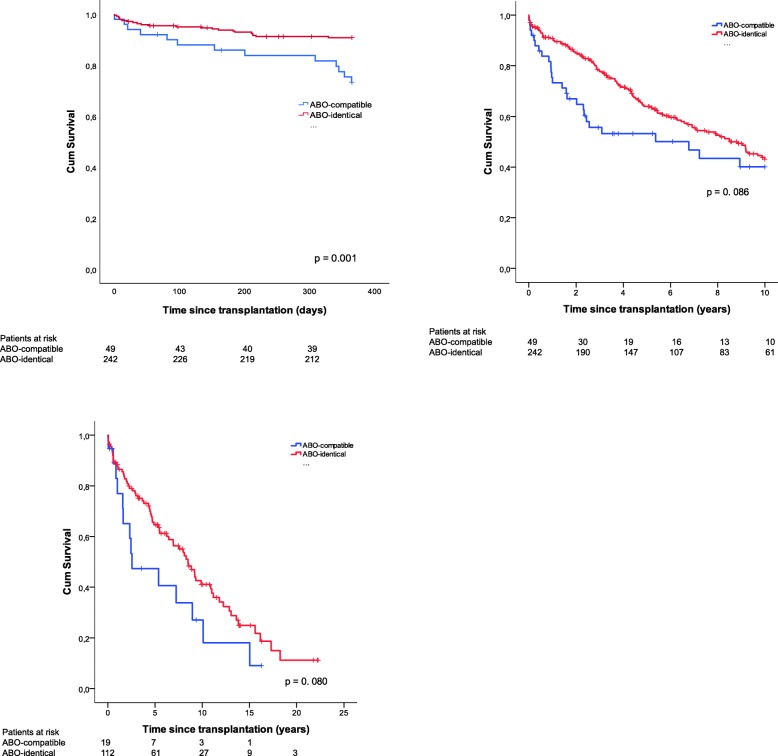
Cumulative retransplantation-free survival for ABO-compatible (N = 49) and ABO-identical (N = 242) LTx for patients with a limited survival up to 1-year (upper left figure), ABO-compatible (N = 49) and ABO-identical LTx (N = 242) up to ten years (upper right figure) and overall survival for ABO-compatible (*N* = 19) and ABO-identical (*N* = 112) LTx in emphysema-patients between 1990 and 2016 (bottom left figure). A significant difference in survival was observed between compatible versus identical matching in patients with limited survival up to 1-year

Cumulative survival estimates for patients with a limited survival up to 1- and 10-years in addition to overall survival for emphysema (COPD+AAT1) patients are illustrated in Fig. 4. Survival rates for recipients with ABO-identical LTx limited up to one year were 95% at 100 days (CI 92–98), 93% at 200 days (CI 90–96), and 91% at 300 days (CI 88–95) compared with recipients with ABO-compatible LTx with 100-, 200-, and 300-day survival rates of 88% (CI 79–97), 86% (CI 76–96), and 84% (CI 73–94), respectively (*p* = 0.001). Regarding patients with a limited survival up to ten-years and overall survival for emphysema patients, no significant difference was found (*p* > 0.05).

### Cox regression

The Cox proportional hazards model (univariable) evaluating ABO-identical vs. ABO-compatible LTx in addition to other risk factors for survival is shown in Table 3. In terms of identical ABO-matching (including interaction of age), gender mismatch, and waiting-list time, no significant difference was found (p > 0.05).

**Table 3:** Cox regression analysis for identical versus compatible ABO-matching, recipient/donor-blood group and other risk factors for retransplantation-free survival (univariable)

	HR	95% CI	*p*-value
Identical ABO-match	0. 803	0. 534–1. 207	0. 291
Age	1. 020	1. 008–1. 033	**0. 002**
Identical ABO-match x age	1. 001	0. 993–1. 009	0. 788
Recipient > 55 years	1. 829	1. 346–2. 486	**0. 001**
BMI	1. 038	1. 001–1. 079	**0. 050**
Male	1. 148	0. 848–1. 556	0. 372
Gender mismatch	1. 130	0. 828–1. 544	0. 441
Waiting list	0. 999	0. 998–1. 000	0. 059
*Infection*			
Recipient CMV	1. 056	0. 718–1. 553	0. 783
CMV-mismatch	1. 290	0. 823–1. 996	0. 253
Recipient EBV	1. 134	0. 815–1. 578	0. 456
EBV-mismatch	1. 655	0. 917–1. 058	2. 985
Recipient toxoplasma	1. 147	0. 791–1. 664	0. 469
Toxoplasma-mismatch	1. 074	0. 648–1. 781	0. 782
*Blood-group (recipient)*			
A			0.782
B	1. 297	0. 790–2. 130	0. 304
AB	1. 072	0. 567–2. 025	0. 830
O	1. 084	0. 774–1. 517	0. 639
*Blood-group (donor)*			
A			0.484
B	1. 290	0. 760–2. 188	0. 345
AB	1. 169	0. 285–4. 794	0. 828
O	1. 292	0. 926–1. 803	0. 131

Entries are bold due to being under 0.05 which are significant results

*CMV* cytomegalovirus, *EBV* Epstein-barr virus, *BMI* body-mass index, *CI* confidence interval, *HR* hazard ratio

Cox regression analyses regarding survival among emphysema-patients is shown in Table 4. Age had a HR of 1.044 (1.010–1.078) and recipients being 55 years or older with a HR of 2.115 (1.306–3.425) (*p* < 0.05). In terms of infection, CMV-mismatching had a HR of 2.588 (1.438–4- 659) and EBV-mismatching a HR of 3.556 (1.511–8.371) (p < 0.05).

**Table 4:** Cox regression analysis for identical versus compatible ABO-matching, recipient/donor-blood group and other risk factors for retransplantion-free survival among emphysema-patients (univariable)

	HR	95% CI	*p*-value
Identical ABO-match	0. 600	0. 336–1. 069	0. 083
Age	1. 044	1. 010–1. 078	**0. 010**
Identical ABO-match x age	0. 995	0. 984–1. 005	0. 328
Recipient > 55 years	2. 115	1. 306–3. 425	**0. 002**
BMI	1. 035	0. 979–1. 094	0. 226
Male	0. 992	0. 632–1. 558	0. 973
Gender mismatch	0. 935	0. 600–1. 459	0. 935
Waiting list	0. 998	0. 997–1. 000	**0. 009**
*Infection*			
Recipient CMV	0. 659	0. 380–1. 144	0. 138
CMV-mismatch	2. 588	1. 438–4- 659	**0. 002**
Recipient EBV	1. 010	0. 634–1. 609	0. 965
EBV-mismatch	3. 556	1. 511–8. 371	**0. 004**
Recipient toxoplasma	1. 254	0. 784–2. 007	0. 345
Toxoplasma-mismatch	1. 426	0. 649–3. 133	0. 377
*Blood-group (recipient)*			
A			0.565
B	0. 950	0. 503–1. 797	0. 875
AB	1. 793	0. 700–4. 595	0. 224
O	0. 897	0. 548–1. 467	0. 665
*Blood-group (donor)*			
A			0.957
B	0. 979	0. 490–1. 955	0. 953
AB	1. 719	0. 233–12. 706	0. 596
O	1. 044	0. 649–1. 677	0. 860

Entries are bold due to being under 0.05 which are significant results

*CMV* cytomegalovirus, *EBV* Epstein-barr virus, *BMI* body-mass index, *CI* confidence interval, *HR* hazard ratio

## Discussion

Lung transplantation is the golden standard of medical intervention for terminally ill patients with end-stage pulmonary disease [13]. Despite the advancements over the years, LTx as a therapeutic intervention is still limited by the scarcity of organs. This significant shortage compels us to find more efficient ways of increasing and managing available donors [14]. In theory, to guarantee the best possible outcome in LTx, organ allocation programs recommend identical antigen-antibody matching between recipient and donor. The survival benefits of ABO-identical matching compared to ABO-compatible matching, however, has been questioned. For HTx patients, ABO-compatible matching shows a worse short-term outcome, but for LTx patients, ABO-compatible matching does not affect short-term survival (from 1 month to 1 year) [15, 16]. Several earlier studies have recommended ABO-matching according to the lung allocation guideline [17] and has been throughout the years been followed strictly by thoracic surgeons at several centers [1, 18]. However, whether there are any long-term benefits are still an issue under debate, including whether it affects long-term survival.

Our study showed no significant differences between ABO-compatible and ABO-identical LTx, in terms of long-term survival. Established risk factors such as gender, age, or height, Tx-type, major indication, and pre-op life support such as ECMO or mechanical ventilation were also evenly distributed in both groups. Use of pre-operative life support in LTx in addition to major indications such as COPD or PH has been shown to be strongly linked with mortality [8]. Among important clinical baselines such as renal, liver, and pulmonary function there were no differences demonstrated between the two groups. This suggests that the current organ allocation program is unbiased and consistent with optimizing best possible outcome despite ABO-matching. An interesting finding emerged when comparing waiting-list time between the two groups, where ABO-identical LTx had 80% longer waiting time than the ABO-compatible group. This suggests a potential to increase the volume of potential donors by accepting ABO-compatible matching, and thereby improving waiting-list survival. Recipients with blood group A, B and AB may hypothetically benefit significantly by including additional ABO-compatible LTx. In contrast, it is important to consider that in theory O-group recipients might be discriminated in such a condition since O-recipients may only accept O-lungs, with the possibility of being deprioritized when higher status is given to the remaining blood groups [19]. Further studies are needed to conclusively determine whether individual blood groups influence outcome in LTx.

Cause of mortality did not differ between ABO-identical and ABO-compatible groups. This is in accordance with Taghavi et al. [20] who found that post-operative outcome does not significantly differ between ABO-compatible and ABO-identical LTx. Earlier studies have reported the feared complication of passenger leucocyte syndrome among ABO-compatible LTx, resulting in the reaction of acute hemolysis between recipient erythrocytes and donor lymphocytes which could prove fatal after LTx [3, 6]. This could not be evaluated in this report, thus further investigation is required.

When stratifying recipients cause of death on donor blood group, no significant differences were found. This is in accordance with Bergenfeldt et al. [21] where no significant difference was shown between donor blood group and cause of death (such as graft failure, infection, malignancy, or miscellaneous). Blood group antigens are found among others on the glycoprotein von Willebrand factor, which varies in amount in accordance with type of ABO-blood group [22]. In general, higher levels are expressed among group AB in contrast to group O showing least amount of the glycoprotein. Von Willebrand factor has been shown to be associated with increased neutrophil activity and ischemia/reperfusion-injury, while liver-Tx has been linked with negative post-operative outcome [23, 24]. However, our study did not support such a premise. As no favorable clinical effect was shown between donor blood group or ABO-identical matching in our study, we can assume that the importance of these factors is limited.

To the authors knowledge, this single-center study has the longest patient follow-up regarding long-term survival comparing ABO-identical vs. ABO-compatible LTx. No survival benefit was found for ABO-identical LTx in the entire cohort, and when excluding SLTx. This is in accordance with Taghavi et al. [20]. The same pattern emerged when dividing our cohort into two different time periods, transplanted before or after 2005. In another model, we tested our null-hypothesis by comparing ABO-matching for emphysema patients. COPD and AAT1 tend to have a higher mortality as these recipients usually undergo SLTx and are often older patients with comorbidities, such as cardiovascular diseases [25]. Even in this group, no benefit was found in ABO-identical matching. An incidental finding was made among emphysema-patients regarding CMV- and EBV-mismatching, as these patients had over 1.5 and 2.5 times higher risk for mortality. These analyses, however, were not adjusted for covariates that could affect survival. Emphysema-patients tend to have a higher age and more comorbidities that could negatively affect outcome.

Body Mass Index, age, and the age group “55 years or older” are known risk factors for mortality in LTx, and this was also found in the present study [15, 26]. On the other hand, no significant interaction between recipient age and identical ABO-matching was found. This suggests that ABO-compatible LTx stands as a promising option without significantly interfering with the outcome for recipients in higher age group. In HTx, however, it has been reported that transplantation outcome could be affected by this interaction as younger recipients tend to have lower frequency of infections and consequently higher frequency of rejections [12]. Further study is needed regarding this interaction in LTx.

This non-randomized retrospective cohort study has several unavoidable limitations [27]. Over the last decades, significant changes have been made in the area of transplantation. Different time-periods in LTx may have different survival outcome affected by factors such as surgical/anesthesia technique, management in the ICU, and introduction of new pharmaceuticals for immunosuppression and infection prevention. Some relevant variables were not obtained, which could influence the results for ABO-compatible LTx. For example, erythrocyte transfusions after transplant procedure and the occurrence of haemolytic anemia were not available. A main limitation in this study is the relatively small number of recipients and the discrepancy in size between the ABO-compatible and ABO-identical LTx groups. A larger dataset and equally large groups could give a more powerful analysis and limit the occurrence of type II errors.

## Conclusions

This single center report has the longest follow-up regarding survival for ABO-compatible vs. ABO-identical matching in LTx and found no difference in long-term survival. In addition, CMV- and EBV-mismatch between recipient and donor for emphysema-patients affect survival negatively. This group, however, does often represent patients of a higher age group with comorbidities and often undergo SLTx, giving these recipients a particular disadvantage in terms of survival. We discovered no survival benefit for ABO-identical LTx in long-term survival. The same pattern followed in the sub analyses when comparing time-periods before and after 2005, excluding SLTx recipients, recipients with up to ten-year limited survival, and even among emphysema-patients. The group with identical matching had over 80% longer time on the waiting list than the group with compatible matching. We see a potential in the use of ABO-compatible LTx to optimize the allocation of donor organs and minimize recipient waiting time.

## Abbreviations

6MWT: 6-min walking test
AAT1: Alpha 1-antitrypsin deficiency
ALT: Alanine transaminase
AST: Aspartate transaminase
ATG: Anti-thymocyte globulin
BMI: Body-mass index
CF: Cystic fibrosis
CLAD: Chronic lung allograft dysfunction
CMV: Cytomegalovirus
COPD: Chronic obstructive pulmonary disease
DLTx: Double-lung transplantation
EBV: Epstein-barr virus
ECMO: Extracorporeal membrane oxygenation
FEV1: Forced volume expiratory capacity 1 s
FVC: Forced volume vital capacity
GVHD: Graft-vs-host disease
HLTx: Heart-lung transplantation
HTx: Heart transplantation
LTx: Lung transplantation
PF: Pulmonary fibrosis
PGD: Primary graft dysfunction
PH: Pulmonary hypertension
Re-LTx: Re-lung transplantation
SLTx: Single-lung transplantation
